# Characterization of Sustainable Asphalt Mixtures Containing High Reclaimed Asphalt and Steel Slag

**DOI:** 10.3390/ma14174938

**Published:** 2021-08-30

**Authors:** Panos Georgiou, Andreas Loizos

**Affiliations:** Laboratory of Pavement Engineering, National Technical University of Athens (NTUA), 9 Iroon Polytechniou Str., 15780 Zografou, Greece; aloizos@central.ntua.gr

**Keywords:** sustainable asphalt mixtures, reclaimed asphalt, steel slag, warm mix asphalt, performance-based properties, moisture susceptibility, rutting resistance, stiffness modulus, fracture resistance, surface macrotexture

## Abstract

Policymakers are implementing the transition to a circular model in all economic sectors to drastically mitigate the effects of climate change. In this regard, the producers of paving products should promote the reuse and recycling of solid waste in the design of sustainable asphalt materials. This study evaluated the performance-based properties of three highly recycled mixtures for wearing courses of asphalt pavements containing steel slag and varying amounts (25, 40, and 50%) of fractionated reclaimed asphalt (RA). In addition, the mixtures incorporated a warm mix asphalt (WMA) organic additive to lower production temperatures compared to a reference hot mix asphalt (HMA). Based on the experimental results, the warm recycled asphalt mixtures show equivalent or better performance compared to HMA in terms of moisture susceptibility, stiffness, rutting and fracture resistance, and surface macrotexture. Therefore, the combined incorporation of RA and steel slag with WMA was proven to be an effective option in designing environmentally friendly and high-performance wearing course mixtures.

## 1. Introduction

Anthropogenic pollution and greenhouse gas emissions are increasing global warming and severely changing ecosystems and climate patterns. Countries have debated approaches to combat the potential implications of these impacts since the early 1990s. These negotiations have produced several significant accords, including the Kyoto Protocol and the Paris Agreement [1]. The European Green Deal is Europe’s more recent response to mitigating climate change. It comprises an overarching policy to achieve a carbon-neutral and sustainable economy by 2050 [2,3]. According to the European Environment Agency, greenhouse gas emissions will continue to decline, although at a slower pace than needed. Therefore, a tremendous effort will be necessary to achieve the higher emission reduction target of 55% proposed for 2030 and the net-zero emissions target by 2050. For this purpose, transitioning from a linear economy to the circular economy concept should be at the forefront of policies for many sectors. This includes the road infrastructure sector, which should strive to provide appropriate solutions for making road pavements more sustainable [4,5].

In this respect, replacing the “end-of-life” concept with the concept of reducing, reusing, and recycling materials, in conjunction with the inclusion of waste streams in production processes, should be methodically undertaken with the aim of consolidating the circular economy approach among pavement producers. This is of utmost importance, considering that annual waste generation will increase by 70% by 2050 [2]. In recent decades, a significant trend has been observed concerning recycling or reusing solid waste materials in highway pavements, which can be categorized into pavement by-products, industrial by-products, and waste produced by the construction and demolition of buildings and road infrastructure [6,7,8,9]. For instance, electric arc furnace steel slag, one of the main by-products of the metallurgical industry, has been utilized in recent decades for substituting coarse aggregates in asphalt mixtures. Previous concerns about the use of steel slag, regarding potential issues such as the impact of leaching or volume expansion under wet conditions on the mixtures’ performance, have been appropriately addressed in recent years through the pre-treatment and production processes [10,11]. Thus, steel slag has been proven to be an effective alternative for the replacement of virgin anti-skid aggregates, taking into consideration its inherent advantageous physical and mechanical properties [11,12].

Moreover, reclaimed asphalt has been effectively and widely used as a valuable component of asphalt mixtures, offering significant economic and environmental benefits, namely raw material conservation, reduction of landfill overburden pressures, and lower fuel usage, energy consumption, and pollutant emissions [6,13]. RA incorporation in new bituminous mixtures has increased since its inception. At contents of up to 15%, RA does not have significant effects on the resultant mixtures. However, at higher contents, studies have shown that RA significantly affects the mixtures’ performance [14,15]. In particular, the properties of aged RA bitumen, and the degree of blending and diffusion achieved between virgin and RA bitumen, increase the potential to reduce cracking and moisture resistance (and, to a lesser extent, rutting resistance). Hence, road authorities remain reluctant to hinder the increased use of high proportions of reclaimed asphalt, particularly in surface course mixtures [14]. This issue may be addressed by coupling RA with recycling agents and warm mix asphalt technologies [16,17,18].

Recycling or rejuvenating agents are usually applied to restore the RA binder proper-ties in a state that resembles that of virgin asphalt binder. Rejuvenators can originate from different sources and are categorized into plant oils, waste-derived oils, engineered products, and traditional and non-traditional refinery base oils [19]. Usually, they are applied through mixing with the aged binder at high temperatures. Selecting the appropriate type should ensure that the rejuvenator not only diffuses rapidly and mobilizes the oxidized RA binder, but also alters the binder rheological properties related to the mixtures’ cracking resistance [20]. Rejuvenator dosage is also crucial to improve the properties of the oxidized binder and, in turn, the recycled mixture’s performance. For instance, the use of a higher quantity of rejuvenator may weaken the adhesion and cohesion between the mixture’s aggregate particles, and thus leading to moisture-related damage and raveling.

Warm mix asphalt is a rapidly expanding technology that allows the mixing and compaction temperature of the asphalt mixtures to be reduced. Temperature reduction is achieved by employing techniques that involve the use of organic additives, chemical additives, and foaming processes (either water based or water containing) [21,22]. These categories differ in terms of the mechanism through which they produce WMA. Organic additives, usually waxes (natural or synthetic) or fatty acid amides, can be added either to the mixture or, more usually, to the bitumen, to lower the bitumen viscosity and improve lubrication, and thus increase the workability of the compacted mix. The underlying mechanism relies on the concept that specific waxes have melting points below the conventional HMA production temperatures; hence, they become dispersible in the mix during the WMA production process, acting as flow modifiers that reduce the bitumen viscosity. On the contrary, the chemical additives, including emulsification agents, surfactants, additives, and polymers, do not change the bitumen viscosity, but act as surfactants to regulate and reduce the frictional forces at the microscopic interface of the aggregates and the bitumen at lower temperatures than those associated with HMA. Temperature reduction achieved using this technique depends on the product and dosage used. In the foaming processes, the underlying mechanism is based on the expansion of water after the transition from a liquid to a vapor state. By adding water to the hot bitumen, a latent steam in the form of foam is generated. This temporarily increases the volume and the surface area of the bitumen, which eventually improves aggregate coating and mixture compaction at lower temperatures. The manner in which water is added to the hot bitumen can vary. In general, these involve either the use of special nozzles (water-based process); or the use of synthetic zeolites, which are composed of aluminosilicates of alkali metals that are hydro-thermally crystallized, to produce the foaming process (water-containing process). Employing the aforementioned techniques produces asphalt at temperatures 20–40 °C lower in comparison to HMA. This results in reduced energy consumption, fewer emissions, and better working conditions.

Beyond the significant social and environmental benefits associated with its use, WMA enables the incorporation of higher contents of reclaimed asphalt materials into the mixtures [22,23]. Multiple studies have investigated the combined use of high RA and WMA on mixture performance. Based on the literature, the warm recycled mixtures show rutting resistance resulting from the high stiffness of RA binder, but also improved cracking resistance due to the reduced secondary ageing of RA binder during mixture production as a result of their lower production temperature. In addition, RA is considered to be water resistant; hence, the increased addition counterbalances the moisture sensitivity of WMA mixtures to produce asphalt mixtures that are more resilient to moisture damage [23,24,25,26,27].

It is noteworthy that a vast number of research studies have focused on producing dense-graded asphalt mixtures, mostly for structural pavement layers, and have investigated the influences of both high RA and WMA on mixtures’ performance. On the contrary, the anti-skid wearing course mixtures have received limited, if any, attention and research effort, despite these mixtures undergoing more frequent M&R. This aspect should raise more awareness in terms of sustainability. In addition, the combined incorporation of RA and steel slag with WMA, which further boosts the circular economy and sustainability concepts, may potentially result in anti-skid wearing course mixtures with superior performance [28]. Therefore, it is vital to investigate the design of longer-life and more durable mixtures for these courses to enhance pavement sustainability.

## 2. Objective

The main objective of this study was to evaluate a wearing course mixture type that incorporated high RA content (25, 40, and 50%), steel slag, and a WMA technology. The effect of higher RA content and WMA on the performance of these mixtures in terms of moisture susceptibility, rutting, stiffness, cracking, and texture was evaluated and compared with a reference hot mix asphalt.

## 3. Materials

The target mixture of this study was a semi-open graded asphalt concrete (AC) with a nominal maximum aggregate size of 12.5 mm, which is commonly used in wearing courses of Greek motorways. Three highly recycled mixtures were designed in the laboratory with steel slag and varying amounts of fractionated RA (25, 40, and 50% by mass), and incorporating an organic WMA bitumen additive for lowering the production temperatures (designated hereafter as WMA-25RA, WMA-40RA, and WMA-50RA). It should be noted that the sum of the steel slag and RA fractions reached as high as 85–95% (by weight) of the mixtures’ aggregate composition, thus demonstrating that these mixtures can be considered to be almost fully recycled mixtures. Metallurgical slag, namely EAF steel slag, which is an artificial aggregate industrially produced in steelworks during ferrous scrap melting at a temperature of 1500 °C combined with lime and other additives, was used as a substitute for high-quality coarse aggregates. After cooling and proper processing, EAF steel slag presents insignificant to no volume expansion, in addition to advantageous physical and mechanical properties, including resistance to fragmentation, surface wear by abrasion, and the polishing effect of vehicle tires. The main properties of the used EAF steel slag aggregates are presented in Table 1, as per EN 13043. The remaining fraction of the mixtures’ composition comprised limestone sand. A fourth mixture with steel slag and limestone sand was also designed and produced at conventional temperatures (designated hereafter as HMA), for benchmarking purposes.

**Figure 1:**
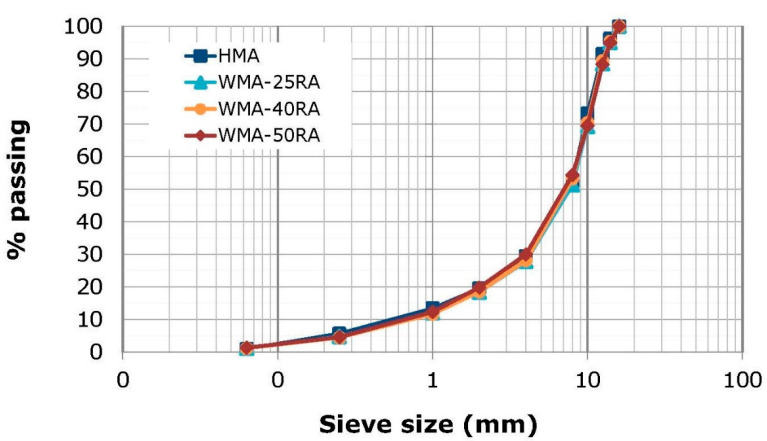
Grading of HMA and WMA-RA mixtures.

All mixtures had almost the same grading, as shown in Figure 1, and optimum bitumen content 4.8% by aggregate weight, and the design air voids ranged between 9 and 10.5%. Various forms of polymer modified bitumen (PmB), designated in terms of their penetration range at 25 °C (e.g., 25/55 pen) and softening point lower limit value (e.g., ≥75 °C) according to EN 14023, were used. Specifically, a PmB graded as 25/55-75 was used for the HMA mixture, whereas a softer PmB graded as 45/80-65 was used for the WMA-RA mixtures to compensate for the aged bitumen of RA materials. To lower the production temperatures of recycled mixtures relative to the HMA, the latter bitumen was mixed with a synthetic paraffin wax additive, at the percentage of 1.5% (by weight of virgin binder), manufactured from natural gas using the Fischer–Tropsch method, which is completely soluble in bitumen above 90 °C and significantly reduces the viscosity of the resulting bitumen blend (i.e., WMA bitumen). For each mixture, loose samples were prepared using laboratory planetary equipment. After mixing, the loose materials of HMA and WMA-RA mixtures were short-term conditioned for 4 and 2 h, respectively, at the planned respective compaction temperatures of 160 and 130 °C. More detailed information about the mixture properties and specimen preparation can be found in a study published by Georgiou and Loizos [28].

## 4. Test Methods for Characterizing Asphalt Mixtures

To fully characterize the mixtures’ performance, both mechanical and functional properties were evaluated, namely, moisture susceptibility, rutting resistance, stiffness modulus, fracture resistance, and surface macrotexture. For specimen fabrication, a steel-segmented roller compactor was used, which is believed to realistically reproduce the field compaction conditions and enables accurate assessment of the on-site characteristics of field compacted asphalt mixtures [29,30,31]. Laboratory specimens were compacted with the aim of achieving a 97% degree of compaction (corresponding to 12–13% air voids).

**Figure 2:**
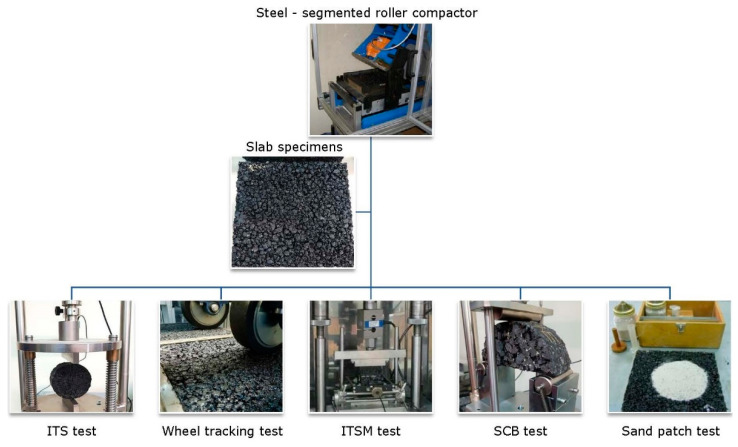
Experimental plan.

Figure 2 illustrates the experimental test plan of this study.

The characterization of the moisture susceptibility of the asphalt mixtures was performed according to EN 12697-12. Two different sets of specimens were prepared: dry and wet specimens. Dry specimens were maintained in a dry state at room temperature, whereas the wet specimens were subjected to vacuum in a pycnometer at 20 °C and kept for 30 min under an absolute pressure of 6.7 kPa, preceded by being saturated and stored in a water bath at 40 °C over a period of 68–72 h. Then, the two sets of specimens were tested at 25 °C for indirect tensile strength (ITS). A strain-controlled loading of 50 mm/min was applied to each sample along with the thickness, according to the EN 12697-23. The maximum load-carrying capacity of each sample was recorded and the indirect tensile strength was calculated along with the ratio of the ITS of wet (water-conditioned) specimens to that of dry specimens (hereafter TSR). TSR is the most popular test method for characterizing the moisture sensitivity of asphalt mixtures.

The rutting susceptibility of asphalt mixtures was assessed by employing the wheel tracking test, according to the EN 12697-22 standard. The test procedure consists of applying repeated loadings on two rectangular compacted specimens (with dimensions 30 × 30 × 4 cm^3^) to assess plastic deformations at high temperature and tire pressure. A standard load magnitude of 700 N was repeatedly applied for up to 10,000 cycles and the rut depth was automatically recorded during the loading cycles using the attached LVDT. The test temperature was selected as 60 °C to simulating summer pavement temperatures, which are more detrimental to the mixture’s resistance in permanent deformation. The rutting indicators obtained from this test were the mean rut depth in the air (RD_AIR_), defined as the downward permanent deformation (in mm) of the specimen surface relative to the original surface; the mean proportional rut depth in air (PRD_AIR_), defined as the ratio of the rut to the thickness of the asphalt tested slab; and the wheel tracking slope in the air (WTS_AIR_), calculated on the linear part (between 5000 and 10,000 load cycles) of the rut depth curve.

The stiffness performance of asphalt mixtures was characterized using the Indirect Tensile Stiffness Modulus (ITSM) test. This non-destructive test consists of sinusoidal load pulses applied along the vertical diameter of the specimen. Controlled deformation stiffness tests at a target deformation of 5 μm based on loading rise-time of 124 ms were performed at low and intermediate pavement service temperatures, namely 5 and 20 °C. The final five load pulses, following ten preconditioning pulses, were used for calculating the stiffness modulus, as per EN 12697-26.

Fracture resistance was evaluated by utilizing semi-circular bending (SCB) tests at 25 °C [32,33]. The SCB tests were conducted on half-disk-shaped specimens having a diameter of 150 mm and thickness of 50 mm, and a mid-span artificial notch of 15 ± 1 mm depth. The test was conducted at a constant loading head displacement rate of 50 mm/min, and both vertical load and deformation were recorded continuously. The fracture resistance, and subsequently the cracking potential of mixtures, was evaluated based on the flexibility index (FI). From the load and displacement history recorded during the SCB tests, the following fracture parameters were extracted to calculate the FI as follows [33,34]:

G_f_ = W_f_ / area, (1)

FI = G_f_ × 0.01/ |m|, (2)

where W_f_ = work of fracture (Joules); area = ligament area (the product of the ligament length and the thickness of the specimen); G_f_ = fracture energy (Joules / m^2^); and m = post-peak slope (kN/mm).

The surface macrotexture of the asphalt mixtures was assessed by employing an experimental, practical-oriented approach, as introduced in an earlier study [35]. This approach includes the fabrication of laboratory asphalt slabs using a steel-segmented roller compactor and macrotexture measurements using the typical sand patch test method. Two measurements were performed, namely longitudinal and lateral to the compaction direction, and averaged to obtain the mean texture depth (MTD) value. Based on this approach, good estimates of the field texture depth were achieved, which subsequently enabled optimization of the texture characteristics of the asphalt mixtures during the mix design process.

## 5. Results and Discussion

### 5.1. Moisture Susceptibility

**Figure 3:**
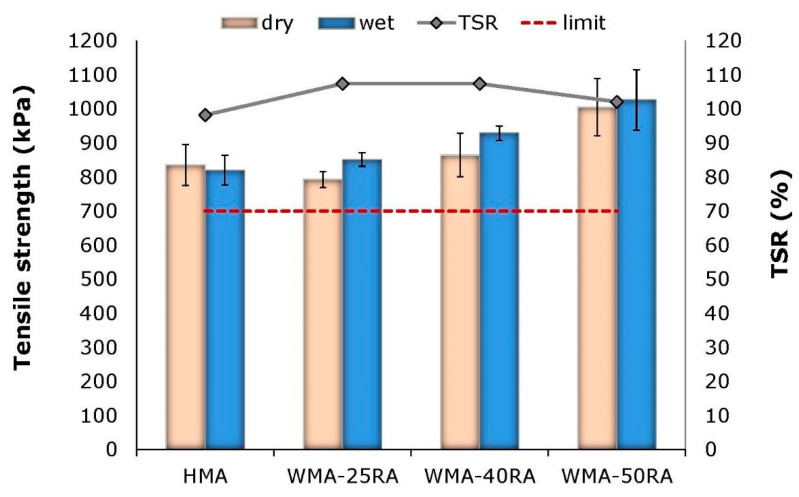
Indirect tensile strength and TSR values.

Figure 3 shows the ITS in wet and dry conditions, in addition to the TSR values, for each mixture.

The results indicate that the mixtures containing RA and steel slag, although produced at a lower temperature, deliver higher tensile strength compared with the reference mix. Adding RA positively influences the moisture resistance of the warm recycled mixtures, as indicated from the increasing wet ITS results. This may be explained in a two-fold manner. First, the increase in the addition of RA results in a higher content of asphalt-coated aggregates, which are insensitive to the effect of water due to the existing bituminous film, regardless of the extent of diffusion and blending of aged RA and virgin bitumen. Second, steel slag is considered to provide strong adhesion in bitumen, due to its physical and chemical properties, and therefore enhances moisture resistance of asphalt mixtures [10]. It is noted that the TSR values in all mixtures distinctly surpass the limit specified in the national specifications. Hence, the studied mixtures exhibit satisfactory performance in terms of moisture resistance.

### 5.2. Rutting Resistance

**Figure 4:**
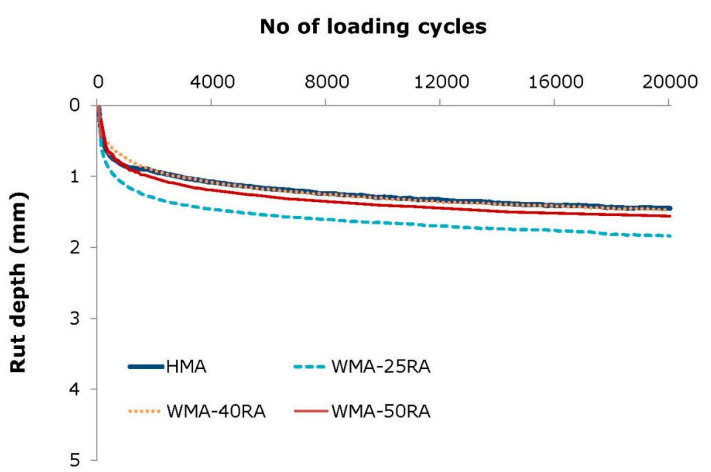
Wheel tracking test results.

The rut depth for all mixtures, as a function of the number of loading cycles, is shown in Figure 4. Moreover, Table 2 shows the corresponding main parameters that characterize their rut resistance at 60 °C.

From the data in Table 2, it is apparent that the warm recycled mixtures, with the exception of WMA-25RA, showed a similar or improved performance compared to HMA in terms of rut resistance, as can be observed by the obtained rut depth (i.e., RD_AIR_), proportional rut depth (i.e., PRD_AIR_), and slope (i.e., WTS_AIR_). The explanation for these results may be related to a better interlock and friction produced from the steel slag in the HMA, and the combined effect of higher stiffness of the blended bitumen combined with the improvement in the particle arrangement of recycled and steel slag aggregates obtained during compaction due to the better workability of the WMA-RA mixtures [36].

**Figure 5:**
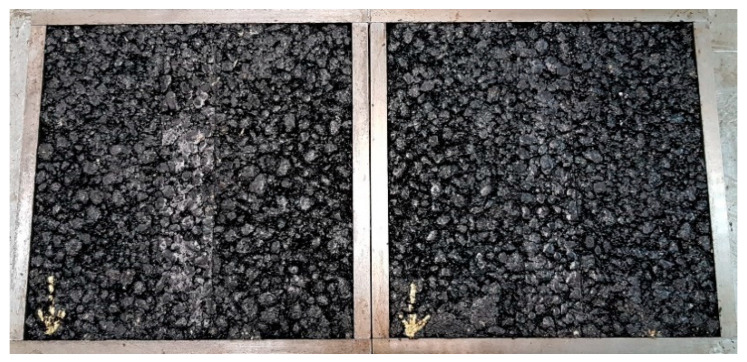
Specimens after wheel tracking test.

It is noteworthy that the rut depth developed in all mixtures was low and limited below 2 mm. This is affirmed in Figure 5, which indicatively illustrates test specimens after being subjected to wheel tracking testing. For this type of bituminous material, for instance, the Greek specification requires that the rut depth at 10,000 cycles should be lower than 7 mm. Therefore, not only the conventional mixture, but also the warm recycled mixtures show acceptable performance and would be particularly suitable for road infrastructures that are vulnerable to plastic deformation, such as those located in warm zone areas.

### 5.3. Stiffness Modulus

**Figure 6:**
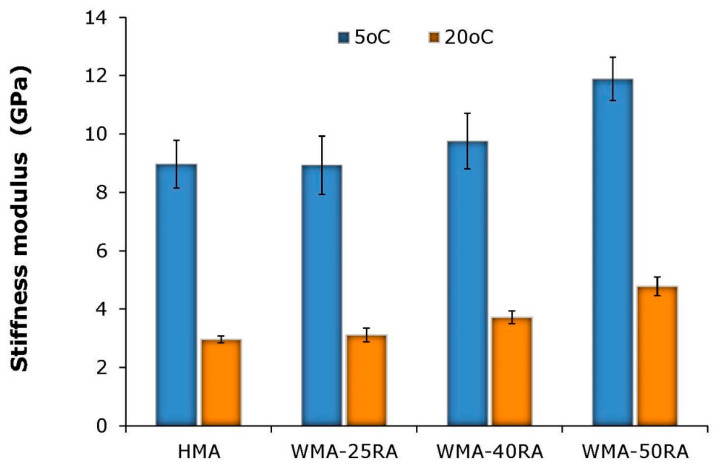
ITSM results.

Figure 6 shows the stiffness modulus test results for all mixtures.

The results indicate that the WMA-RA mixtures exhibit a similar or higher stiffness modulus compared to the conventional HMA mixture. This indicates that the lower production temperatures of recycled mixtures do not adversely affect their stiffness properties. The comparison among the WMA-RA mixtures indicates that the stiffness modulus increases with the increase in RA proportion; however, the increase in stiffness does not correlate linearly with the RA increase. For higher RA content, in particular, it is argued that the stiffness depends both on the stiffening of the aged RA binder, and the extent of blending and diffusion of RA and virgin binders. For instance, at 5 °C, the addition of 25, 40, and 50% RA to the control mix increased the stiffness modulus by −0.4, 8.8, and 32.6%, respectively. Accordingly, at 20 °C, the stiffness increased by 5.1, 25.6, and 61.2%. These results indicate that the combined effect of steel slag and RA on the stiffness modulus is more pronounced at intermediate temperatures than at low temperatures. Moreover, all recycled mixtures, particularly those incorporating 40 and 50% RA, yield moduli values similar or greater than the typical values of dense-graded mixtures [30]. This demonstrates the ability of these mixtures to support traffic-induced stresses, thus contributing to the load-bearing capacity of pavements.

### 5.4. Fracture Resistance

**Figure 7:**
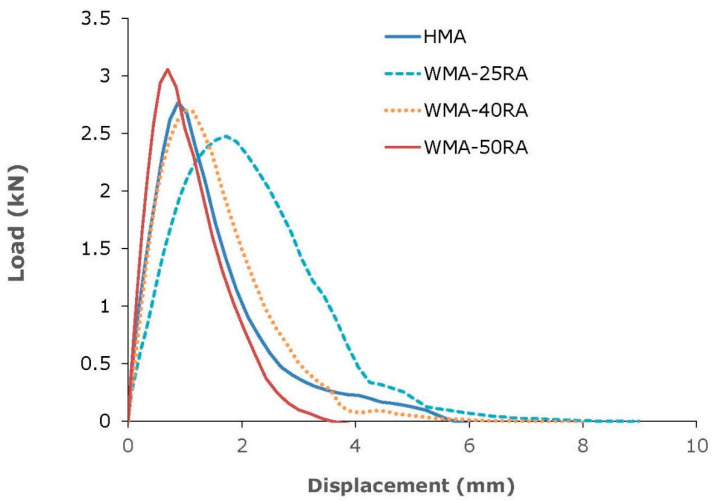
Semi-circular bending test results.

**Figure 8:**
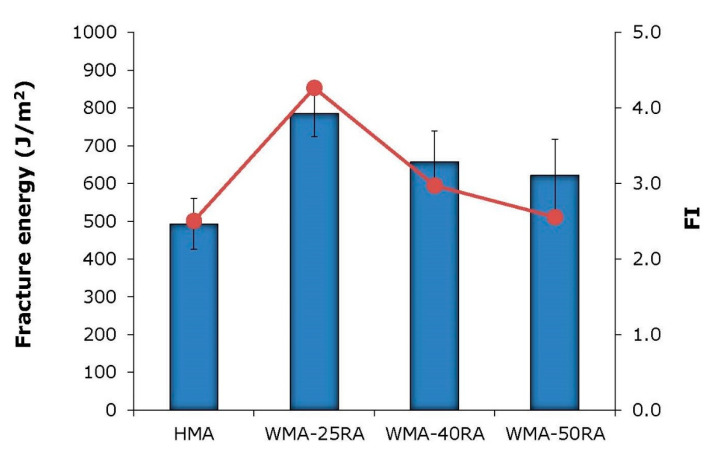
Fracture energy and flexibility index results.

Results of the semi-circular bending tests are shown in Figure 7, and the corresponding fracture energy and flexibility index are shown in Figure 8.

Based on the G_f_ results, the HMA showed the most brittle behavior. G_f_ represents the energy dissipated by the crack propagation; thus, the higher the fracture energy, the better the cracking resistance of the asphalt mixtures. On the contrary, all warm recycled mixtures exhibited higher fracture energy values than the control mix, by 26–59%. Similar findings were derived from the FI results. Regarding the recycled mixtures, the fracture energy decreased with increasing RA content, and the WMA-25RA mixture exhibited superior fracture properties. In addition, the flexibility index decreased with increasing RA, which may be expected because the amount of aged bitumen increased with increasing RA.

Overall, both fracture energy and flexibility index results indicate that the conventional mixture is more prone to cracking compared with the warm recycled mixtures. Therefore, the incorporation of the WMA organic additive, along with the softer bitumen, compensates for the stiffening effect of the aged RA bitumen, thus ensuring the cracking resistance of highly recycled mixtures. Fatigue resistance evaluation testing, supplemented with advanced imaging techniques for capturing the mortar film thickness distribution, may offer a better understanding of the cracking mechanism of HMA and WMA-RA mixtures [37].

### 5.5. Surface Macrotexture

**Figure 9:**
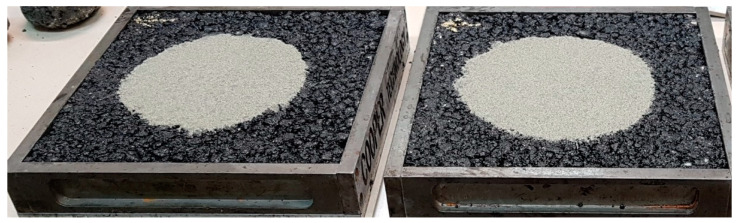
Surface macrotexture characterization.

**Figure 10:**
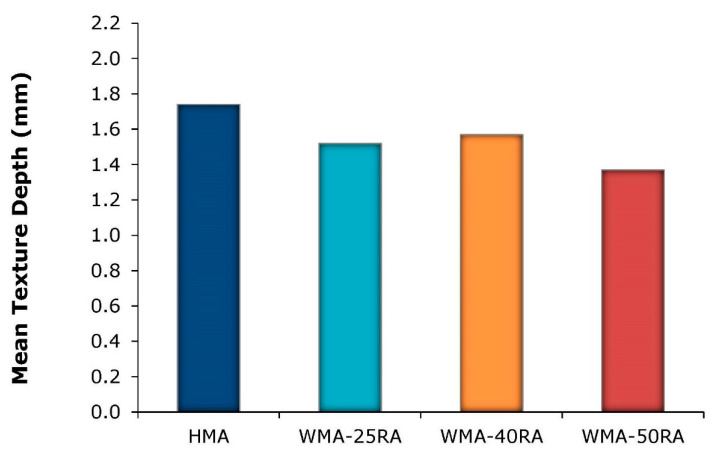
Texture depth results.

Sand patch tests were performed on roller-compacted asphalt slabs, as illustrated in Figure 9. The macrotexture test results of the mixtures studied are shown in Figure 10.

The results indicate that the HMA and the WMA with 40% RA exhibited the highest texture depths. On the contrary, the lowest texture corresponded to the WMA with 50% RA. Taking into consideration that the same compaction energy was applied to all mixtures, the closer texture of some recycled mixtures may be attributed to their better workability and compactibility properties, which resulted in denser aggregate packing [28]. However, it has been demonstrated that the surface macrotexture achieved in the field is also related to the construction techniques, and the steel-segmented roller compaction method enables the efficiency of field compaction modes in achieving satisfactory texture levels to be simulated and evaluated [35]. In this respect, future work will focus on deter-mining the field compaction mode that optimizes the resulting surface texture properties of the studied mixtures.

## 6. Conclusions

This study investigated the performance of anti-skid wearing course mixtures designed with varying high contents of reclaimed asphalt (25, 40, and 50%) and steel slag aggregates, and produced at reduced temperatures using an organic (synthetic wax) additive compared to a reference HMA. Based on the research conducted to characterize their performance-based properties, the following conclusions can be drawn:

The indirect tensile strength of the warm recycled mixtures is improved with the incorporation of steel slag and increased RA. As a result, the TSR values indicate enhanced moisture damage resistance.

The high-temperature performance of all mixtures is almost equivalent, based on the wheel tracking test, and satisfies the requirements prescribed in the technical specifications.

Due to the presence of high RA content, the warm recycled mixtures show stiffness values higher than those of the reference HMA. The stiffening effect of the RA binder was more pronounced when comparatively evaluating the stiffness properties at an intermediate temperature.

Nevertheless, this effect does not significantly compromise the fracture characteristics of the warm recycled mixtures. For example, the mixture with 50% RA was characterized by better fracture resistance compared with that of HMA, based on the SCB test and the I-FIT method.

Both the warm mix asphalt with 40% RA and the HMA were found to offer the optimum texture depth, and thus superior surface characteristics related to road safety issues.

Overall, the characteristics of the WMA with steel slag and 40% RA ensure both a balanced and superior performance to that of the conventional HMA.

Nevertheless, the results of this study generally indicate that high reclaimed asphalt content (up to 50%) and steel slag can be valorized towards developing circular, resource-efficient, and durable anti-skid wearing course mixtures. It was also demonstrated that these mixtures offer significant environmental benefits [38], and therefore can be considered to be a promising sustainable solution for full-scale implementation.

## Figures and Tables

**Table 1:** Main properties of steel slag aggregates.

Properties	Fractions 12/16 mm, 4/12 mm
Grading category	Gc90/10
Volume stability	V_3.5_
Flakiness index	FI_10_
Resistance to fragmentation Resistance to wear	LA_20_ MDE_10_
Resistance to abrasion	AAV_10_
Resistance to polishing	PSV_62_

**Table 2:** Parameters from wheel tracking tests.

Parameters	HMA	WMA-25RA	WMA-40RA	WMA-50RA
	Mean (Standard Deviation)			
Wheel tracking slope (WTS_AIR_) (mm/10^3^ cycles)	0.032 (0.003)	0.037 (0.013)	0.032 (0.0001)	0.030 (0.003)
Proportional rut depth (PRD_AIR_) (%)	3.63 (0.04)	4.60 (0.57)	3.69 (0.02)	3.90 (0.28)
Rut depth (RD_AIR_) (mm)	1.45 (0.01)	1.84 (0.23)	1.48 (0.01)	1.56 (0.11)

## Data Availability

The data presented in this study are available on request from the corresponding author.
